# Volumizing and Cogged Threads for Nose Augmentation

**DOI:** 10.1111/jocd.16542

**Published:** 2024-09-01

**Authors:** Jovian Wan, Youngjin Park, Soo Yeon Park, Kyu‐Ho Yi

**Affiliations:** ^1^ Asia Pacific Aesthetic Academy Hong Kong China; ^2^ Obliv Clinic Incheon Korea; ^3^ Made‐Young Plastic Surgery Clinic Seoul Korea; ^4^ Division in Anatomy and Developmental Biology, Department of Oral Biology, Human Identification Research Institute, BK21 FOUR Project Yonsei University College of Dentistry Seoul Korea; ^5^ Maylin Clinic (Apgujeong) Seoul Korea

**Keywords:** cosmetic technique, nose, rhinoplasty, thread

## Abstract

**Background:**

Nose augmentation using nonsurgical methods, including volumizing and cogged threads, has gained popularity due to its minimally invasive nature, shorter recovery times, and reduced risks compared to traditional rhinoplasty. However, there is limited literature on the use of these techniques in the Asian population.

**Aims:**

This study aims to present two successful cases of nose augmentation using volumizing and cogged threads, providing evidence of their effectiveness and demonstrating the insertion techniques through a supplementary video.

**Patients/Methods:**

Two female patients (aged 26 and 33) underwent nonsurgical nose augmentation. The first patient received volumizing threads to enhance nasal contour, while the second patient received cogged threads to refine the nasal tip and alar base. Both procedures were performed using polydioxanone (PDO) threads, with follow‐ups conducted to assess the outcomes.

**Results:**

Both patients exhibited significant improvement in nasal contour and projection, with outcomes maintained for up to 8 months post‐procedure. The patients reported high satisfaction with the aesthetic results, and no complications were observed during the follow‐up period.

**Conclusions:**

Volumizing and cogged threads offer a promising nonsurgical alternative for nose augmentation, particularly in patients seeking minimal invasiveness and natural‐looking results. While the initial outcomes are positive, further research is needed to evaluate the long‐term safety and effectiveness of these techniques, especially in the Asian demographic.

## 1 Introduction

**FIGURE 1:**
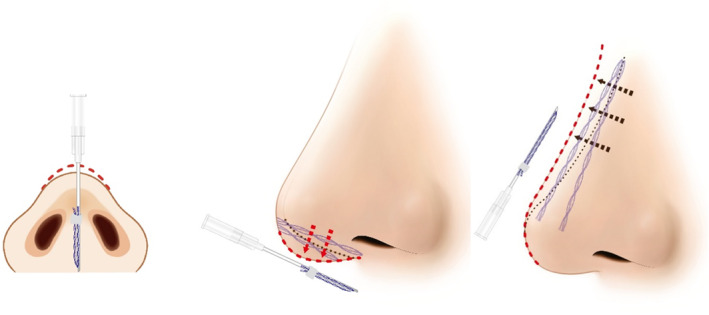
The procedure for nose augmentation using volumizing threads (N‐Scaffold, N‐Finders Co., Ltd, Korea) typically involves the use of longer threads to correct the nasal bridge and the utilization of shorter threads in the nasal tip region for elevation.

Recent advancements in nonsurgical rhinoplasty procedures involve either the combination of volumizing threads with fillers or the exclusive application of volumizing threads. When these threads, designed to support and volumize nasal tissues, are employed, they establish a framework that sustains the nasal bridge and tip, thereby extending procedural efficacy. This approach not only reduces the amount of filler required filler but also helps mitigate the risk of developing an “avatar nose”—characterized by an excessively broad nose due to excessive filler usage. Furthermore, using threads alone can enhance nose contouring, offering advantages such as reduced procedure durations, faster recovery times, and reduced risks associated with fillers [1]. Consequently, nonsurgical rhinoplasty is gaining popularity in Southeast Asia [2]. Besides volumizing threads, cogged threads play a crucial role in nonsurgical rhinoplasty procedures. Practitioners often opt for cogged threads with barbs, although smooth monofilament threads may also be chosen. Longer threads are primarily employed to address the nasal bridge, while shorter threads are specifically applied to elevate the nasal tip (Video S1 and Figure 1) [1].

The first nose reshaping procedure using threads was introduced in 2010 as an office‐based procedure, involving the use of a double‐sided needle with a suture. This method aimed to anchor the lower lateral cartilages to the glabellar periosteum, facilitating nasal tip rotation and projection [3]. In the existing literature, significant emphasis is placed on understanding standardized nose anthropometric points, clinical landmarks, lines, and angles, all of which play crucial roles in achieving desirable outcomes for nose augmentation procedures [4, 5, 6]. However, despite the wealth of information available, there remains a noticeable gap in the literature when it comes to utilizing volumizing and cogged threads alone for nose augmentation within the Asian demographic. While various techniques and approaches have been explored, there is a lack of comprehensive guidance specifically tailored to this demographic, leaving room for further research and exploration in this area.

The aim of this article is to present successful cases of nose augmentation utilizing volumizing and cogged threads, and to demonstrate the thread insertion technique through a Video S1.

## 2 Case Presentation

### 2.1 Case 1

**FIGURE 2:**
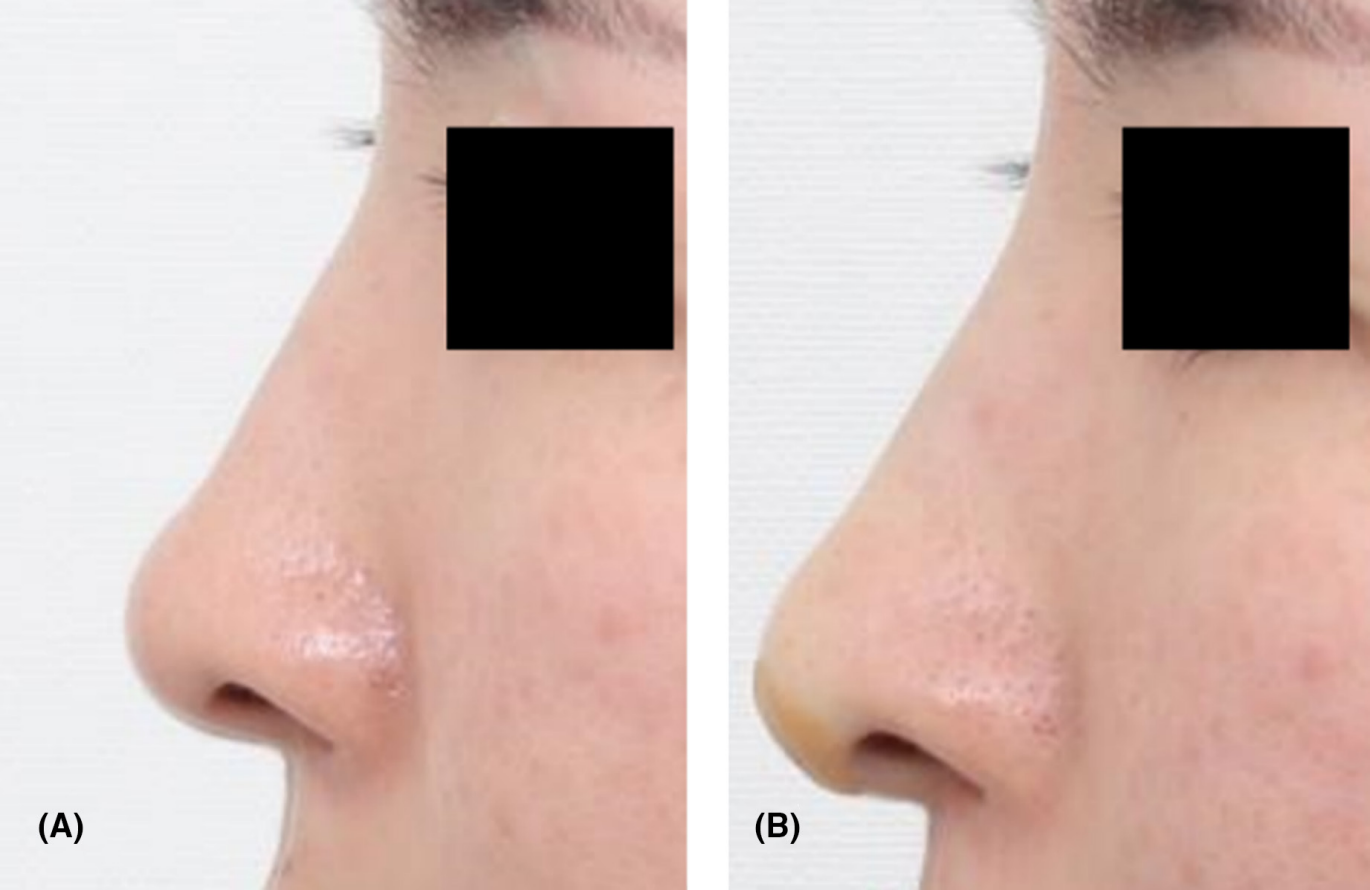
A 26‐year‐old female patient, reluctant to undergo filler treatment but seeking improvement in nasal contour, chose the use of volumizing threads. The profile images display the nasal contour before (A) and after (B) the procedure.

**FIGURE 3:**
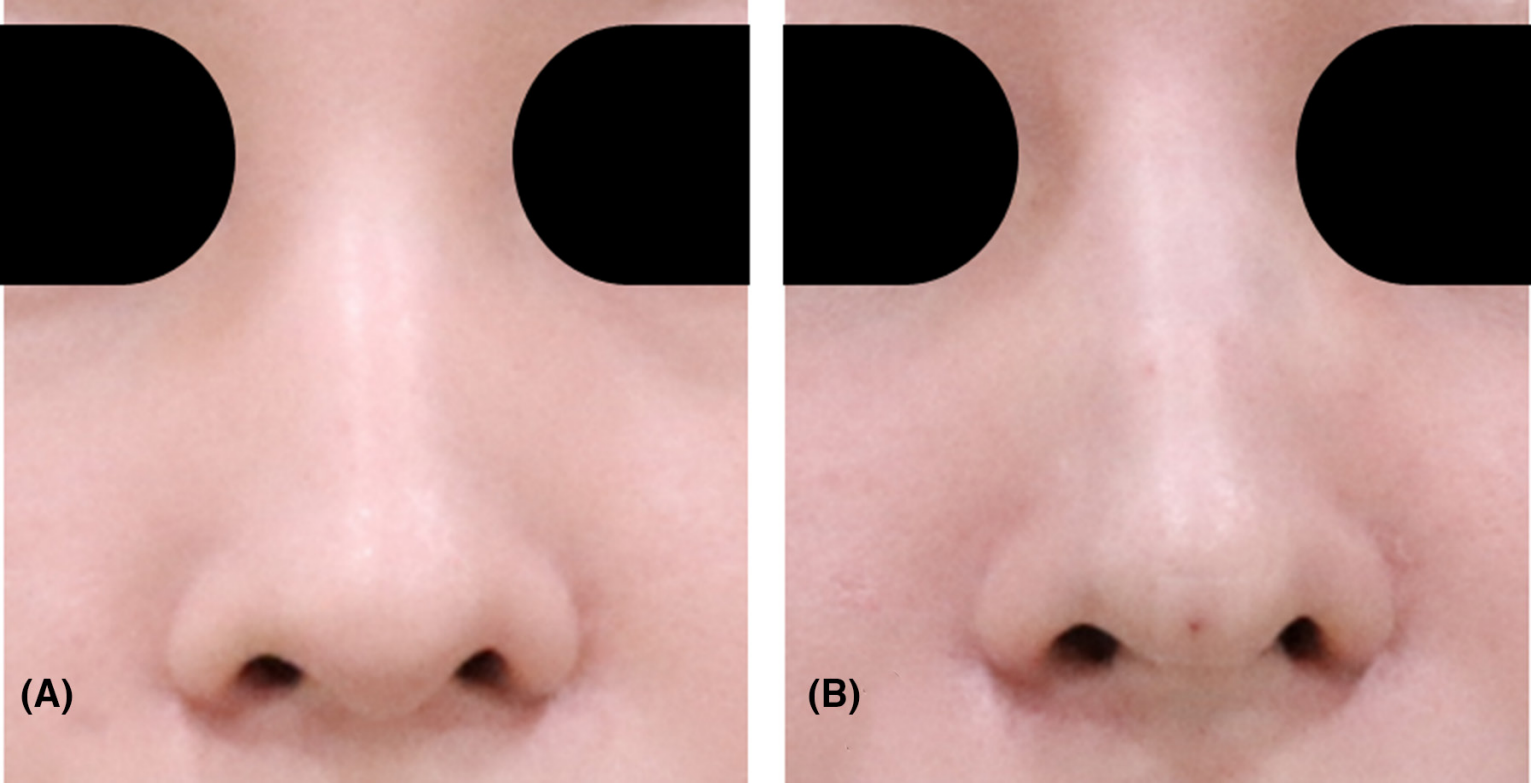
Frontal images showing the before (A) and after (B) results of the procedure from Case 1.

A 26‐year‐old female, apprehensive about filler treatment but desiring an improved nasal contour, opted for treatment involving volumizing threads. Four lines of threads were inserted along each dorsum of the nose for enhancement, with an additional four threads inserted into the columellar area. Following the procedure, notable improvement in nasal contour was observed, and the results were well‐maintained (see Figures 2 and 3). The patient expressed satisfaction with the outcomes, both immediately after the procedure and during the 8‐month follow‐up.

### 2.2 Case 2

**FIGURE 4:**
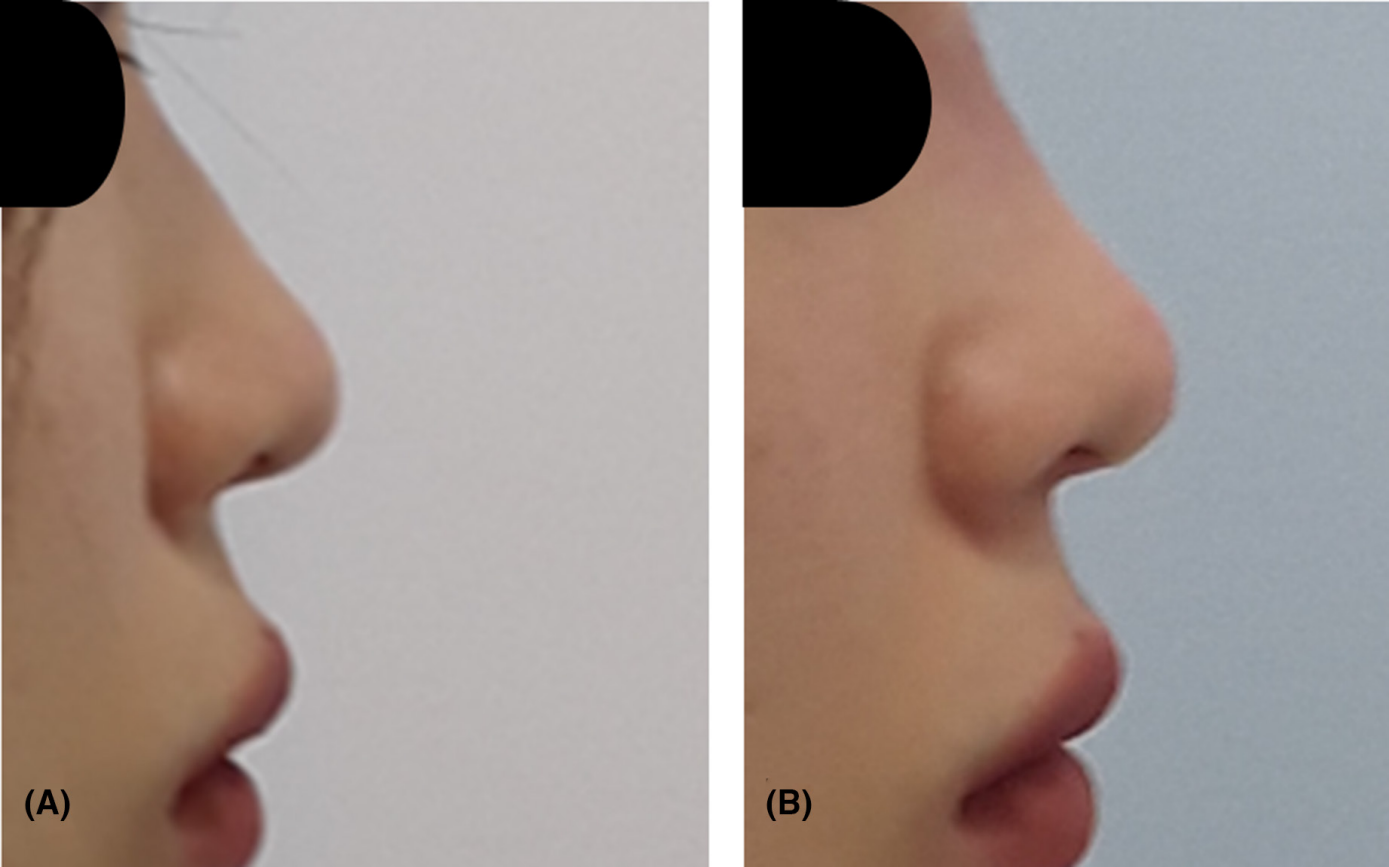
A 33‐year‐old female patient underwent nonsurgical rhinoplasty, involving the insertion of six cogged threads in the columella to enhance tip projection and three cogged threads for alar retraction. The profile images illustrate the nasal appearance before (A) and after (B) the procedure from Case 2.

**FIGURE 5:**
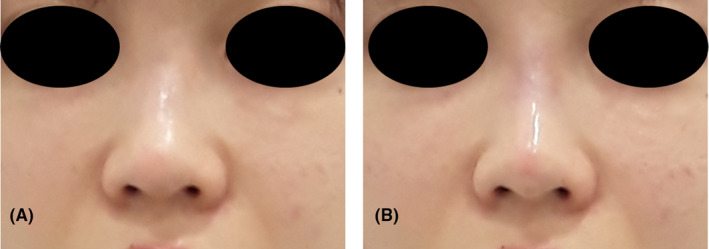
Frontal images depicting the before (A) and after (B) results of the procedure from Case 2.

**FIGURE 6:**
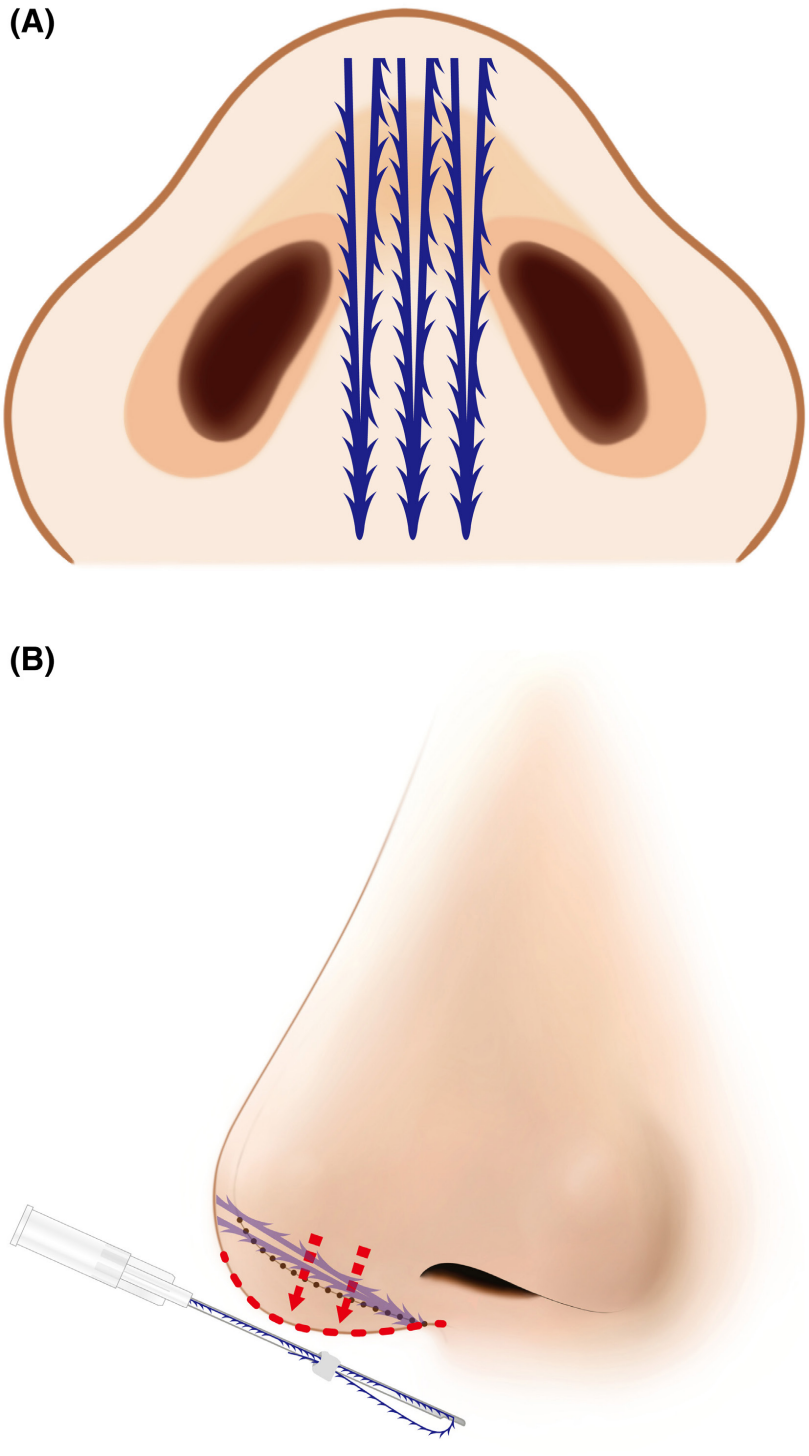
Multidirectional barbed threads used (Y‐Ko, N‐Finders Co., Ltd, Korea) for tissue fixation, with three threads positioned between the nasal tip and maxilla to provide support and structural enhancement. The panel (A) is schematic image from bottom to top and panel (B) is lateral view.

A 33‐year‐old woman sought treatment to refine the appearance of her bulbous nose. Upon examination, her nasal alar base appeared wide, and her nasal tip lacked prominence. She underwent nonsurgical rhinoplasty involving the insertion of six cogged threads in the columella to enhance tip projection and three cogged threads for alar retraction (Figures 4 and 5). Following the procedure, the nasal tip appeared more refined, and the nasal alar base appeared narrower (Figure 6). The patient expressed satisfaction with the results, both immediately after the treatment and during the 8‐month follow‐up period.

## 3 Discussion

Volumizing threads are designed with specific structures, such as multiple fine monofilaments or twisted coil shapes, to provide a cushion‐like support when inserted into the skin. They are also engineered to resist compression and maintain their original form within tissues post‐insertion. These unique characteristics allow volumizing threads to effectively fill voids underneath the skin, address deeply etched wrinkles, and sculpt specific areas of the face to achieve desired aesthetic outcomes [7]. Volumizing threads for nose augmentation hold significant value in the aesthetic market, and authors based in Southeast Asia frequently encounter patients requesting this procedure. Southeast Asian patients often present with distinctive features including a low nasal bridge, low nasal tip, short nose, wide nostrils, and a short columella. Additionally, they tend to have a thick nasal tip skin tends containing dense fibroadipose tissue, while the cartilage in the nostril area is typically small and delicate. This can lead to unsatisfactory outcomes and potential complications, such as implant deviations or protrusions, in invasive rhinoplasty procedures. As a result, nonsurgical rhinoplasty using threads is increasingly sought after in this region as a safer and more tailored alternative to address these specific nasal concerns. Optimal candidates for nose thread augmentation typically lack a surgical history, possess sufficient soft tissue and skin mobility, and exhibit a loose tip area conducive to effective manipulation through pinching [8].

Unlike volumizing threads, cogged threads are employed to redefine and reshape the profile of the nose, offering immediate and effective fixation [8]. These threads feature protrusions designed to anchor into tissues, allowing them to pull and secure sagging tissues against the force of gravity. An important distinction between cogged threads and volumizing threads lies in their direction and depth of insertion. In rhinoplasty procedures using cogged threads, the threads are strategically positioned in various orientations, encompassing tip projection, nose length elongation, alar extension, and augmentation of the columella base. The objective is to achieve symmetrical and proportionate features while preserving the natural contours of the nose [9].

The authors opted for polydiaxonane (PDO) volumizing and cogged threads due to PDO's high elasticity and restoration capabilities. In contrast to nylon or polycaprolactone, PDO threads have demonstrated superior effectiveness in exerting restoration forces [10].

PDO, an absorbable polymer degraded through simple hydrolysis within the body, has emerged as a favored material for facial thread lifting due to its absorbable nature. Lerwick's work [11] in 1983 provided crucial insights into the efficacy and safety of PDO thread lifting, with histopathological examinations confirming the development of fibrous encapsulation tissue around PDO threads. Furthermore, research by Yoon et al. [12] has revealed that PDO threads stimulate collagen formation, leading to the formation of fibrous connective tissue, increased adjacent capillary vessels, and a reduction in fat layer thickness through fat cell denaturation. Recent studies have further supported PDO's role as a neovascularization agent in collagen regeneration [13, 14]. Despite commencing degradation approximately 3 months post‐insertion, PDO threads are completely absorbed within 6 months, yet their collagen‐inducing effects persist for over 10 months [12]. Our study observed that nose augmentation remained evident up to 8 months post‐procedure, although our follow‐up did not extend beyond this period. In our clinical practice, patients treated with volumizing and cogged threads typically undergo annual procedures.

Park et al. [1] have highlighted the potential complications of nonsurgical rhinoplasty with nose threads, stressing the importance of patient selection and proactive management for positive outcomes. Complications include thread protrusion, extrusion, migration, and skin infection, underscoring the need for complete thread removal to prevent further issues such as migration and protrusion through the oral cavity. Similarly, Sulamanidze, Lanfranchi, and Diaspro [15] outlined possible complications of nose thread lifting, such as asymmetry, migration or extrusion of threads, infection, skin irregularities, and inflammation. Irreversible or severe complications are infrequently encountered [10]. Throughout our case study, we observed no complications during the 8‐month follow‐up period.

Park et al. [1] observed that although repeated thread treatments may produce favorable outcomes, excessive interventions can result in challenges such as fibrotic tissue formation. Furthermore, Sulamanidze, Lanfranchi, and Diaspro [15] discovered that the duration of nose augmentation post‐thread insertion depends on the rigidity of the cartilage framework, rather than skin thickness. They also noted a case of dorsal irregularity attributed to the visibility of thread loops, which can be promptly addressed through intervention. Lee [10] suggests incorporating fat grafts with repeated thread procedures to improve the results. This findings underscore the necessity for further investigation into the longer term safety and effectiveness of threads for nose augmentation, encompassing the ideal frequency for repeating procedures. Practitioners typically offer nose volumizing and cogged thread treatments to individuals on a yearly basis in our clinical practice.

## 4 Conclusion

In conclusion, our cases demonstrate the efficacy of nose augmentation using volumizing and cogged PDO threads. The satisfaction of the patients highlights the effectiveness of this intervention in delivering the desired aesthetic outcomes. Despite potential complications, the benefits of PDO threads in nose augmentation present a promising option for minimally invasive nonsurgical rhinoplasty. Further research is necessary to assess the longer term safety and efficacy of PDO volumizing and cogged thread procedures for nose augmentation.

## Author Contributions

**Jovian Wan, Youngjin Park, Soo Yeon Park, and Kyu‐Ho Yi:** conceptualization, writing – original draft preparation, writing – review and editing, and visualization. **Kyu‐Ho Yi:** supervision. All authors have reviewed and approved the article for submission.

## Conflicts of Interest

The authors declared no potential conflicts of interest with respect to the research, authorship, and publication of this article. This study was conducted in compliance with the principles set forth in the Declaration of Helsinki.

## Supporting information


Video S1

## Data Availability

The data that support the findings of this study are available from the corresponding author upon reasonable request.
